# Detection of Novel SARS-like and Other Coronaviruses in Bats from Kenya

**DOI:** 10.3201/eid1503.081013

**Published:** 2009-03

**Authors:** Suxiang Tong, Christina Conrardy, Susan Ruone, Ivan V. Kuzmin, Xiling Guo, Ying Tao, Michael Niezgoda, Lia Haynes, Bernard Agwanda, Robert F. Breiman, Larry J. Anderson, Charles E. Rupprecht

**Affiliations:** Centers for Disease Control and Prevention, Atlanta, Georgia, USA (S. Tong, C. Conrardy, S. Ruone, I.V. Kuzmin, Y. Tao, M. Niezagoda, L. Haynes, L.J. Anderson, C.E. Rupprecht); Jiangsu Provincial Center for Disease Control and Prevention, Nanjing, People’s Republic of China (X. Guo); National Museum, Kenya Wildlife Service, Nairobi, Kenya (B. Agwanda); Centers for Disease Control and Prevention Kenya, Nairobi (R.F. Briman)

**Keywords:** Coronavirus, SARS, bat viruses, pathogen discovery, emerging viral infections, dispatch

## Abstract

Diverse coronaviruses have been identified in bats from several continents but not from Africa. We identified group 1 and 2 coronaviruses in bats in Kenya, including SARS-related coronaviruses. The sequence diversity suggests that bats are well-established reservoirs for and likely sources of coronaviruses for many species, including humans.

The 2003 outbreak of severe acute respiratory syndrome (SARS) generated renewed interest in coronaviruses (CoV) and the source for the SARS CoV that caused the outbreak in humans (*1*). Serologic studies demonstrated that the virus had not previously circulated in human populations to any large extent and suggested a source of zoonotic origin (*2**–**4*). A likely natural viral reservoir for the virus was not identified until horseshoe bats (*Rhinolophus* spp.) in several regions in the People’s Republic of China were demonstrated to harbor SARS-like CoVs (*5*). Subsequently, a number of other SARS-like CoVs, as well as CoVs from antigenic groups I and II, were identified from bats in Asia, Europe, and North America, and coronavirus antibodies were detected in African bat species (*6**–**11*). It is not surprising that a growing number of CoVs have been detected in bats. To date, >60 viral species have been detected in bats because their biodiversity (second only to rodents), high population densities, wide distribution, and ability to fly over long distances allow them to harbor and easily spread multiple infectious agents. Bats have long been known as natural hosts for lyssaviruses and more recently have been recognized as potential reservoirs for emerging human pathogens, including Ebola, Marburg, Nipah, and Hendra viruses in addition to SARS-CoV (*12**,**13*). 

## The Study

**Figure 1:**
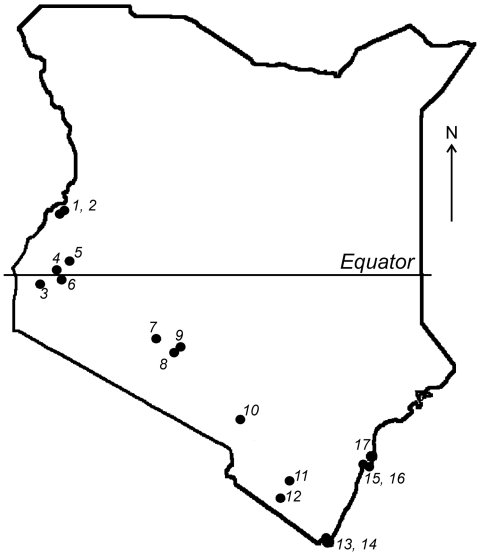
Map of Kenya showing the locations of 17 bat collection sites.

Given the association of bats with emerging infectious diseases, field surveys were performed during July–August 2006 in the southern portion of Kenya (Figure 1). The selection of sites was based on preliminary data regarding bat roost locations and observations of bats in the field during the survey. Attempts were made to collect specimens from 10–20 animals of each species present in each location. Bats were captured manually and by using mist nets and hand nets; adults and subadults of both sexes were captured. Each bat was measured, sexed, and identified to the genus or species level when possible. Blood samples and oral and fecal swabs were collected; the animals were then euthanized in compliance with field protocol. Blood, fecal swabs, and selected tissue samples were transported on dry ice from the field and stored at –80°C.

Fecal swabs (n = 221; Table) were screened for the presence of CoV RNA using 2 semi-nested reverse transcription–PCR (RT-PCR) assays. For the pan-coronavirus RT-PCR, conserved primers were designed from highly conserved regions of the RNA-dependent RNA polymerase (RdRp) gene 1b based on available CoV sequences (1st and 2nd round forward 5′-ATGGGITGGGAY TATCCWAARTGTG-3′; 1st round reverse 5′-AATTAT ARCAIACAACISYRTCRTCA-3′; 2nd round reverse 5′-CTAGTICCACCIGGYTTWANRTA-3′). For the pan–bat coronavirus RT-PCR, conserved primers were designed from the same highly conserved regions based on available bat CoV sequences and presumed to be more specific to bat coronaviruses (1st and 2nd round forward 5′-ATGGGITGGGAYTATCCWAARTGTG-3′; 1st round reverse 5′-TATTATARCAIACIACRCCATCRTC-3′; 2nd round reverse 5′-CTGGTICCACCI GGYTTNACRTA-3′). Total nucleic acids were extracted from 200 μL of a phosphate buffered saline suspension of each swab by using the QIAamp Mini Viral Elute kit (QIAGEN, Santa Clarita, CA, USA), according to the manufacturer’s instructions. The seminested RT-PCR was performed by using the SuperScript III One-Step RT-PCR kit and Platinum Tag Kit (Invitrogen, San Diego, CA, USA). The positive PCR products were purified by gel extraction by using the QIAquick Gel Extraction kit (QIAGEN) according to the manufacturer’s instructions; they were then sequenced on an ABI Prism 3130 automated sequencer (Applied Biosystems, Foster City, CA, USA), according to the manufacturer’s instructions.

**Figure 2:**
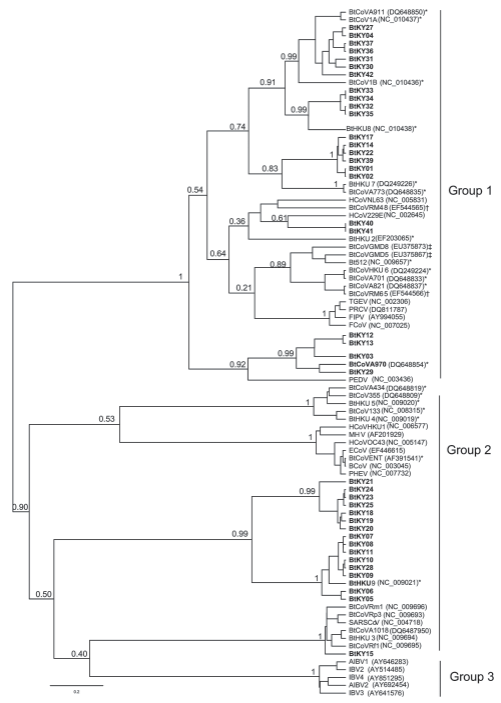
Phylogenetic tree generated using Bayesian Markov Chain Monte Carlo analysis implemented in Bayesian Evolutionary Analysis Sampling Trees (BEAST; http://beast.bio.ed.ac.uk) by using a 121-nt fragment of the RdRp gene 1b from 39 coronaviruses (CoVs) in bats from Kenya. CoVs from this study are shown in **boldface**; an additional 47 selected human and animal coronaviruses from the National Center for Biotechnology Information database are included. The Bayesian posterior probabilities were given for deeper nodes. CoV groups (1 to 3) based on International Committee on Taxonomy of Viruses recommendation are indicated. Bat coronaviruses from the People’s Republic of China (*), northern Germany (†), and North America (‡) are labeled. Scale bar indicates number of nucleotide substitutions per site.

Of 221 bat fecal swabs examined, 41 (19%) were positive by at least 1 of the 2 seminested RT-PCR assays (Table). One specimen had 2 distinct CoV sequences, each amplified by 1 of the 2 PCR assays, giving a total of 42 distinct CoV sequences. To characterize the overall diversity of CoV sequences, in this study a phylogenetic tree (Figure 2) of the 121-bp fragment of RdRp was generated from 39 coronaviruses from bats in Kenya and 47 selected human and animal coronaviruses from the National Center for Biotechnology Information database based on the Bayesian Monte Carlo Markov Chain method (*14*). Three of the 42 sequences were not of sufficiently high quality to include in this tree. Some nodes had low Bayesian posterior probabilities (Figure 2). Longer sequences from these viruses are needed to refine their phylogenetic relationships.

**Table:** Results of detection of CoV RNA in fecal swabs of bats from Kenya*

Bat species	Geographic location	PCR results, no. positive/no. tested	Clusters
*Cardioderma cor*	15	0/10	
	12	1/3	BtCoVA970-like
*Chaerophon* sp*.*	6	1/14	BtHKU7-like
	17	6/19	BtHKU7-like, BtKY18-like, SARSCoV-like
	3	0/5	
*Chaerophon pumilus*	3	2/3	HCoV229E-like
	11	0/4	
*Coleura afra*	11	0/1	
	14	0/1	
*Eidolon helvum*	4	6/10	BtKY18-like
*Epomophorus wahlbergi*	9	0/3	
*Hipposideros commersoni*	14	1/10	BtHKU9-like
*Hipposideros ruber*	2	0/4	
	5	0/2	
*Lissonycteris angolensis*	5	0/10	
*Miniopterus africanus*	10	1/8	BtCoV1A-like
*Miniopterus inflatus*	5	7/12	BtCoV1A-like, BtHKU8-like
*Miniopterus minor*	13	1/16	BtCoV1A-like
*Miniopterus natalensis*	1	1/7	BtCoV1A-like
*Neoromicia tenuipinnis*	6	0/4	
*Otomops martinsseni*	7	2/19	BtHKU7-like
*Pipistrellus* sp*.*	8	0/1	
*Rhinolophus hildebrandtii*	10	0/4	
*Rhinolophus* sp*.*	14	0/1	
	13	0/1	
	8	0/5	
*Rousettus aegyptiacus*	1	2/10	BtKY18-like
	2	2/9	BtCoVA970-like, BtHKU9-like
	16	6/9	BtCoVA970-like, BtHKU9-like
	13	2/11	BtHKU9-like
*Taphozous hildegardeae*	14	0/3	
*Taphozous* sp*.*	11	0/2	
Total		41/221 (19%)	

*CoV, coronavirus; SARS, severe acute respiratory syndrome.

Among the 39 sequences in the tree, 23 belonged to previously defined group 1 and were mapped into 5 different sequence clusters. The 121-bp sequences in these 5 clusters had an average nucleic acid (NA) sequence identity of 88%, 85%, 81%, 77%, and 80% when compared with the next closest previously characterized CoVs (i.e., BtCoV1A, BtHKU8, BtHKU7, HCoV229E, and BtCoVA970, respectively). The remaining 16 sequences would likely be placed into group 2. Two sequences from *Chaerophon* spp. bats (location 17) were closely related to a SARS-like CoV cluster, including 1 sequence shown in Figure 2 (BtKY15) and another (BtKY16) that was 1 of the 3 low-quality sequences excluded from the tree. These 2 NA sequences show ≈89% identity with the nearest previously characterized bat: SARS-like CoV, BtCoVRF1, shows ≈80% NA sequence identity to SARS CoV (Urbani strain) and ≈63% NA sequence identity to the human group 2 CoV HCoVOC43. The 15 remaining NA sequences were grouped into 2 clusters. One cluster contains the recently described BtHKU9 with >95% NA sequence identity, and the other cluster (BtKY18-like cluster) contains no other previously known CoVs, with <75% NA sequence identity to BtHKU9.

The pattern of CoV detections by bat species and location demonstrates several features concerning coronaviruses in bats. A given bat species in the same location can harbor several distinct CoVs as noted for *Chaerophon* spp. (location 17), *Miniopterus inflatus* (location 5), and *Rousettus aegyptiacus* (location 2 and 16); similar CoVs can also been seen in the same type of bat in different locations, as noted for BtCoV1A-like cluster CoVs being detected in *Miniopterus* spp. bats of 4 species from different locations. One *M. inflatus* bat from location 5 harbored 2 different, but closely related, CoVs, 1 (BtCoV 36) from the BtCoV1A-like cluster and 1 (BtCoV 35) from the BtHKU8-like cluster (Figure 2). CoVs of these 2 closely related clusters were detected in *Miniopterus* spp. bats, but not detected in other bat genera, including those that shared roosts with *Miniopterus* spp. bats. This finding is consistent with studies from China in which BtCoV1A-like and BtHKU8-like CoVs were frequently identified but only in *Miniopterus* spp. bats (*15*). This may suggest that viruses of the BtCoV1A-like cluster and the BtHKU8-like cluster are specifically adapted to *Miniopterus* spp. bats and not easily transmitted to other bat species.

In contrast, other genetically similar CoVs were detected in several different bat species. For example, CoVs from th BtHKU7-like cluster were detected in both *Chaerophon* spp. and *Otomops martinsseni* bats; CoVs from the BtCoVA970-like cluster were detected in *Cardioderma cor* and *Rousettus aegyptiacus* bats; CoVs from the BtKY18-like cluster were detected in *Chaerophon* spp., *Eidolon helvum*, and *R. aegyptiacus* bats; and CoVs from the BtHKU9-like cluster were detected in *Hipposidereos commersoni* and *R. aegyptiacus* bats.

## Conclusions

These data demonstrate that the CoV diversity in bats previously detected in Asia, Europe, and North America is also present, possibly to a greater extent, in Africa. The extent of this diversity among CoVs may be shown more clearly through additional studies in bats, and increased demonstration of CoV diversity in bats may require a reconsideration of how they should be grouped. The frequency and diversity of CoV detections in bats, now in multiple continents, demonstrate that bats are likely an important source for introduction into other species globally. Understanding the extent and diversity of CoV infection in bats provides a foundation for detecting new disease introductions that may, like SARS, present a public health threat.
